# Supplementary material for: Empirical methods for controlling false positives and estimating confidence in ChIP-Seq peaks
Source: BMC Bioinformatics. 2008 Dec 5;9:523. doi: 10.1186/1471-2105-9-523 (PMC2628906; doi:10.1186/1471-2105-9-523)
Supplement: Additional File 1 — A variety of html documents from the USeq web site detailing the available applications, their best usage, output file type descriptions, command line menus, etc. [file 1471-2105-9-523-S1.zip › USeqUserGuides/JavaDoc.zip!/JavaDoc/edu/utah/seq/parsers/Tag2Point.html]

Tag2Point


|  |  |  |
| --- | --- | --- |
| **Overview** \| **Package** \| **Class** \| **Use** \| **Tree** \| **Deprecated** \| **Index** \| **Help** | |  |
| **PREV CLASS**   NEXT CLASS | **FRAMES**    **NO FRAMES**     **All Classes** |  |
| SUMMARY: NESTED \| FIELD \| CONSTR \| METHOD | DETAIL: FIELD \| CONSTR \| METHOD |  |


---


## edu.utah.seq.parsers Class Tag2Point

```
java.lang.Object
  edu.utah.seq.parsers.Tag2Point
```

---

``` public class Tag2Point extends java.lang.Object ```

Splits a tag file (chr start stop ... strand, ie xxx.bed) file by chromosome and strand.
For each region a single hit is assigned to the
center position. Files are saved using the bar format.
Final positions are in interbase coordinates.

**Author:**
:   david.nix@hci.utah.edu

---

**Constructor Summary**

`Tag2Point(java.lang.String[] args)`


| **Method Summary** | |
| --- | --- |
| `int` | `calculateMiddle(java.lang.String[] tokens)`             Using interbase coordinates so length = end - start. |
| `boolean` | `convertTempFiles()` |
| `PointData` | `loadPositionValues(int[] sortedPositions)` |
| `static void` | `main(java.lang.String[] args)` |
| `static void` | `printDocs()` |
| `void` | `processArgs(java.lang.String[] args)`             This method will process each argument and assign new varibles |
| `boolean` | `splitTagToTemp()`             Splits a tag file by chromosome and strand to seperate files. |

**Methods inherited from class java.lang.Object**

`equals, getClass, hashCode, notify, notifyAll, toString, wait, wait, wait`

**Constructor Detail**

### Tag2Point

```
public Tag2Point(java.lang.String[] args)
```


**Method Detail**

### convertTempFiles

```
public boolean convertTempFiles()
```

---


### loadPositionValues

```
public PointData loadPositionValues(int[] sortedPositions)
```

---


### splitTagToTemp

```
public boolean splitTagToTemp()
```

:   Splits a tag file by chromosome and strand to seperate files.

---


### calculateMiddle

```
public int calculateMiddle(java.lang.String[] tokens)
                    throws java.lang.Exception
```

:   Using interbase coordinates so length = end - start.

    :   **Throws:**: `java.lang.Exception`

---


### main

```
public static void main(java.lang.String[] args)
```

---


### processArgs

```
public void processArgs(java.lang.String[] args)
```

:   This method will process each argument and assign new varibles

---


### printDocs

```
public static void printDocs()
```


---


|  |  |  |
| --- | --- | --- |
| **Overview** \| **Package** \| **Class** \| **Use** \| **Tree** \| **Deprecated** \| **Index** \| **Help** | |  |
| **PREV CLASS**   NEXT CLASS | **FRAMES**    **NO FRAMES**     **All Classes** |  |
| SUMMARY: NESTED \| FIELD \| CONSTR \| METHOD | DETAIL: FIELD \| CONSTR \| METHOD |  |


---
